# Performance of TDDFT Vertical Excitation Energies of Core‐Substituted Naphthalene Diimides

**DOI:** 10.1002/jcc.26188

**Published:** 2020-03-06

**Authors:** Ayush K. Narsaria, Julian D. Ruijter, Trevor A. Hamlin, Andreas W. Ehlers, Célia Fonseca Guerra, Koop Lammertsma, F. Matthias Bickelhaupt

**Affiliations:** ^1^ Department of Theoretical Chemistry and Amsterdam Center for Multiscale Modeling (ACMM) Vrije Universiteit Amsterdam Amsterdam The Netherlands; ^2^ Van't Hoff Institute for Molecular Sciences University of Amsterdam Amsterdam The Netherlands; ^3^ Department of Chemistry University of Johannesburg Johannesburg South Africa; ^4^ Leiden Institute of Chemistry, Gorlaeus Laboratories Leiden University Leiden The Netherlands; ^5^ Institute of Molecules and Materials Radboud University Nijmegen The Netherlands

**Keywords:** charge‐transfer excitations, density functional calculations, naphthalene diimides, solvent effects, time‐dependent density functional theory

## Abstract

We have evaluated the performance of various density functionals, covering generalized gradient approximation (GGA), global hybrid (GH) and range‐separated hybrid (RSH), using time dependent density functional theory (TDDFT) for computing vertical excitation energies against experimental absorption maximum (λ_max_) for a set of 10 different core‐substituted naphthalene diimides (cNDI) recorded in dichloromethane. The computed excitation in case of GH PBE0 is most accurate while the trend is most systematic with RSH LCY‐BLYP compared to λ_max_. We highlight the importance of including solvent effects for optimal agreement with the λ_max_. Increasing the basis set size from TZ2P to QZ4P has a negligible influence on the computed excitation energies. Notably, RSH CAMY‐B3LYP gave the least error for charge‐transfer excitation. The poorest agreement with λ_max_ is obtained with semi‐local GGA functionals. Use of the optimally‐tuned RSH LCY‐BLYP* is not recommended because of the high computational cost and marginal improvement in results.

## Graphical abstract

We have evaluated the performance of various exchange‐correlation functionals for reproducing the experimental absorption maximum in core‐substituted naphthalene diimides through quantum‐chemical TDDFT and statistical calculations. Results are analyzed and an appropriate TDDFT protocol has been developed.

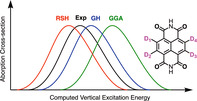

## 1 INTRODUCTION

In recent years, organic dyes have found applications that extend far beyond their traditional use such as in optoelectronic devices ranging from light emitting diodes^1^, ^2^, ^3^, ^4^, ^5^ to dye sensitized solar cells.^6^, ^7^, ^8^ Theoretical methods offer substantial time and resource savings in the design and tuning of the absorption properties of dyes for optimized performance in optoelectronic applications, compared to the standard trial and error approach in the laboratory.

Time‐dependent density functional theory (TDDFT) has become the method of choice for calculating the absorption properties of relatively large chromophores due to its balance between accuracy and computational expense.^9^, ^10^, ^11^, ^12^, ^13^, ^14^, ^15^ However, many chromophores or dyes used in optoelectronic devices show charge‐transfer (CT) behavior^16^ or have highly delocalized excitations^17^ for which TDDFT with semi‐local or local exchange‐correlation functionals inherently fails. The reason for this deficiency is often attributed to the lack of the integer discontinuity in these types of functionals.^18^, ^19^, ^20^ In the Kohn‐Sham framework, both the occupied and virtual orbitals “feel” an effective field of N–1 electrons. The virtual orbitals can therefore be seen as approximations to excitation energies as long as the electron and hole are in close proximity.^21^, ^22^ However, this is not the case in CT excitations where the electron and hole are separated in space. The local character of the exchange hole leads to an unphysical stabilization of the CT excitation. These shortcomings can be alleviated partially by using global hybrid (GH)^23^ and largely by range‐separated hybrid (RSH) functionals.^24^, ^25^, ^26^

**Figure 1:**
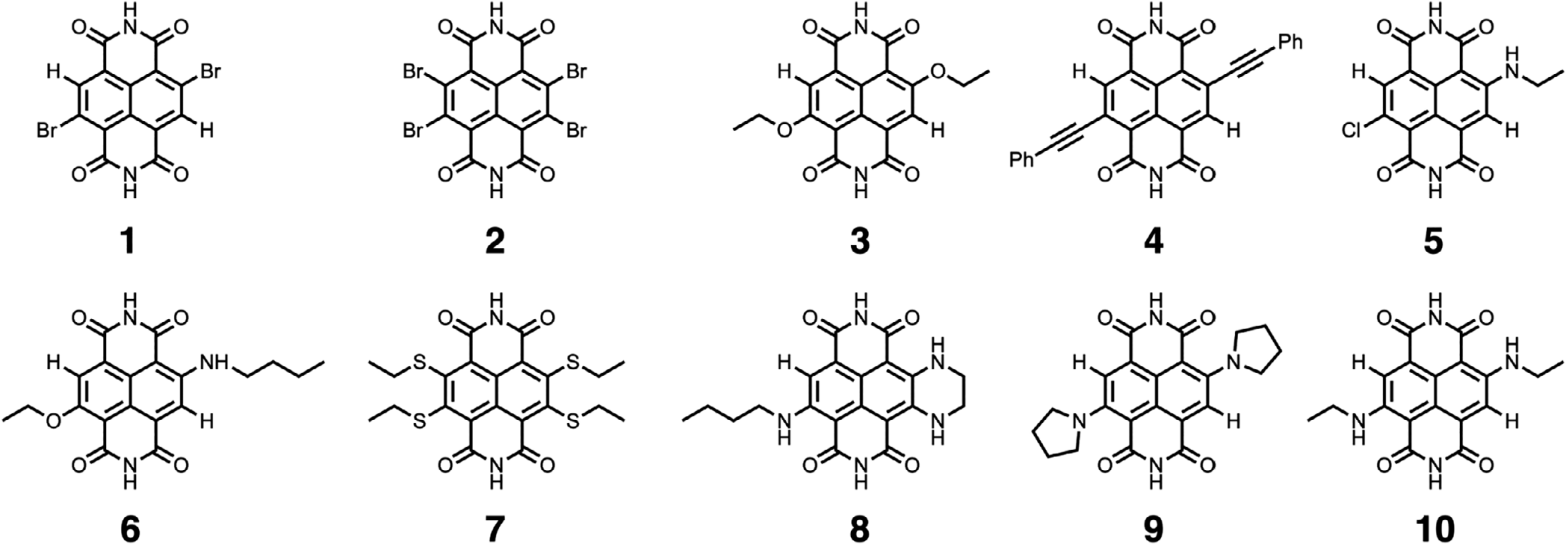
Structures of the selected cNDIs for the TDDFT benchmark (**1–** **10**)

In this paper we present a systematic TDDFT study of core‐substituted naphthalene diimides (cNDIs) which are well known for their tunable absorption and emission properties, ranging over the entire visible to near‐infrared spectrum.^27^, ^28^, ^29^, ^30^, ^31^, ^32^ The parent NDI molecule has been extensively used as an electron acceptor exhibiting a high π‐acidity,^33^, ^34^ as well as for electron transport^35^, ^36^ and photoinduced CT applications.^37^, ^38^, ^39^ An accurate quantum computational approach is indispensable to gain a comprehensive understanding of these excited‐state processes. To this end, we have computed the lowest dipole‐allowed singlet electronic excitation energies for 10 different core‐substituted naphthalenediimides (cNDI; shown in Figure 1) in the gas‐ (*E* ^vert‐abso^) and in the condensed‐phase (*E* ^vert‐abso^[DCM]) using linear‐response TDDFT and compared these to experimental UV/Vis absorption maximum λ_max_ values recorded in dichloromethane (DCM).^40^ The calculations have been performed with a selection of generalized gradient approximation (GGA), global hybrid (GH), range‐separated hybrid (RSH) and optimally‐tuned range‐separated hybrid (OT‐RSH) functionals. Note that the computed vertical excitation energies and the measured absorption maximum λ_max_ are not physically equivalent, although they are often directly compared and do agree remarkably well.^14^, ^41^, ^42^ A more correct assessment would require comparing the computed 0–0 transition energy *E*_0‐__0_ to the measured absorption‐fluorescence crossing point.^43^ However, due to the lack of well‐resolved vibronic spectra, the computed excitation energies are compared against λ_max_.

## 2 COMPUTATIONAL DETAILS

### 2.1 General procedure

All calculations were carried out with the Amsterdam density functional (ADF2013) program.^44^, ^45^, ^46^ Equilibrium geometries were optimized using the BP86 functional.^47^ The BP86 functional is one of the three best DFT functionals for the accuracy of geometries, with an estimated unsigned error of 0.008 Å.^48^ In all cases, the we used the TZ2P basis set, which is created from a large uncontracted set of Slater‐type orbitals of triple‐ζ quality, augmented by two sets of polarization functions (d and f on heavy atoms; 2p and 3d sets on H). Core electrons (e.g., 1s for second period, 1s2s2p for third period, 1s2s2p3s3p for fourth period, 1s2s2p3s3p3d4s4p for fifth period, and 1s2s2p3s3p3d4s4p4d for sixth period) were treated by the frozen core approximation. We used the pair‐atomic density fitting to calculate the Coulomb and exchange terms in the self‐consistent field (SCF) procedure. Scalar relativistic corrections were included self‐consistently using the zeroth‐order regular approximation (ZORA).^49^ All geometry optimizations were performed in the gas‐phase on isolated molecules. All the equilibrium geometries were verified by vibrational analyses to be (local) minimum energy structures (zero imaginary frequencies). The frontier orbital wavefunctions exhibit a nodal plane at the two nitrogen positions in the diimide ring, which suggests that the alkyl or phenyl groups at these positions would have no significant impact on the molecular electronic properties.^32^, ^50^ We found exactly the same observation. For example, the lowest dipole‐allowed vertical excitation energy of N,N‐DIP_2_‐**1** is blue‐shifted by only 0.05 eV when both phenyl rings are removed from the diimide positions and replaced by hydrogens (see Figure S1 and Table S1). Therefore, to reduce the computational effort the substituents at the diimide position were replaced with hydrogens in all the systems (**1–10**).

### 2.2 Vertical excitation energies

The singlet vertical excitation energies were computed with TDDFT^51^ for our model cNDI systems mimicking gas‐phase and dichloromethane (DCM) solvent conditions. The effect of DCM was included using the nonequilibrium conductor‐like screening model (COSMO) in the linear‐response framework.^52^ The various functionals employed were GGAs BLYP,^53^, ^54^, ^55^ and OLYP,^56^ GHs B3LYP with 20% exact exchange,^57^ and PBE0 with 25% exact exchange,^58^ and RSHs LCY‐BLYP (0% and 100% exact exchange at short‐ and long‐range, respectively) and CAMY‐B3LYP (19% and 65% exact exchange at short‐ and long‐range, respectively).^59^ The TZ2P basis set was used for the computations. The BLYP functional was used to test the basis set convergence for the excitation energies. Increasing the basis set from TZ2P to a larger QZ4P basis set had no significant influence on the vertical excitation energies (see Table S2). The RSH functional splits the Coulomb operator into a short‐ and long‐range interaction part attenuated by a switching function. The most common type of switching function is the complementary error function erfc, due to the straightforward computation of integrals involving the error function and Gaussian basis functions. However, there is no computational advantage in combination with Slater orbitals. Therefore, the exponential function exp(−γ*r*_12_), called the Yukawa potential,^58^ in combination with the Coulomb operator have been implemented in ADF. The γ parameter determines how rapidly the switching occurs between the short‐range (SR) and long‐range (LR) term [Equation (1)].

(1) $$\frac{1}{r_{12}}=\mathrm{SR}+\mathrm{LR}=\frac{1-[\alpha +\beta (1-\exp\left(-\gamma r_{12}\right)]}{r_{12}}+\frac{\alpha +\beta (1-\exp\left(-\gamma r_{12}\right)}{r_{12}}$$

The parameters *α* and the sum *α + β* denote the fraction of exact exchange at short and long interelectronic distance *r*_12_, respectively. For example, on using the RSH functional LCY‐BLYP with the empirically‐derived parameters γ = 0.75, *α* = 0.00 and *β* = 1.00 the exact exchange and semi‐local DFT exchange vanishes completely at *r*_12_ = 0 and *r*_12_ = ∞, respectively. Similarly, the second RSH CAMY‐B3LYP functional has the parameters of γ = 0.34, *α* = 0.19, and *β* = 0.46. We also evaluated the performance of OT‐RSH LC‐BLYP*, where * represents ab‐initio tuned range‐separation parameter γ in LC‐BLYP based on the IP theorem of DFT,^60^, ^61^, ^62^, ^63^ details of which are provided in the next section.

### 2.3 Calculating the optimal range‐separation parameter

It is well known that the fraction of exact and DFT exchange along with the range‐separation parameter γ employed in RSHs are system‐dependent.^64^ Invoking the IP theorem of DFT, the optimally tuned value of γ can be computed in an ab‐initio manner for each system. The IP theorem states that the energy of the highest occupied molecular orbital ε(N) for an N‐electron system should be equal to the negative of the ionization potential of the N‐electron system –IP(N), calculated as the energy difference *E*(N–1) – *E*(N).^60^, ^61^, ^62^, ^63^ Approximate XC functionals lead to a large deviation between ε(N) and IP(N), and so the optimally‐tuned formalism tries to minimize this error such that ε(N) = –IP(N) is satisfied to the best possible extent. This formalism can also be applied to the N + 1 system, such that ε(N + 1) = *E*(N) – *E*(N + 1) = –IP(N + 1) equals the electron affinity of the N‐electron system EA(N). Numerous studies have shown that OT‐RSH functionals can vastly improve the electronic spectra along with other response properties.^65^, ^66^, ^67^, ^68^, ^69^, ^70^ Thus, following [Equations 1 and 2] we performed ab‐initio tuning of the switching parameter γ in the LCY‐BLYP functional by minimizing the *J*(γ) function with respect to γ for each molecule in the benchmark set.

(2) $$J\left(\gamma \right)=-\overset{}{\sum_{i=0}^{1}\left[\varepsilon_{H}\left(N+i\right)+\mathrm{IP}\left(N+i\right)\right]}$$

Condensed‐phase tuning leads to too small γ values, which would reintroduce the delocalization error leading to underestimation of the excitation energies for the solvated molecules.^71^ For this reason, all tuning calculations were performed on isolated molecules mimicking gas‐phase conditions.

## 3 RESULTS AND DISCUSSION

**Figure 2:**
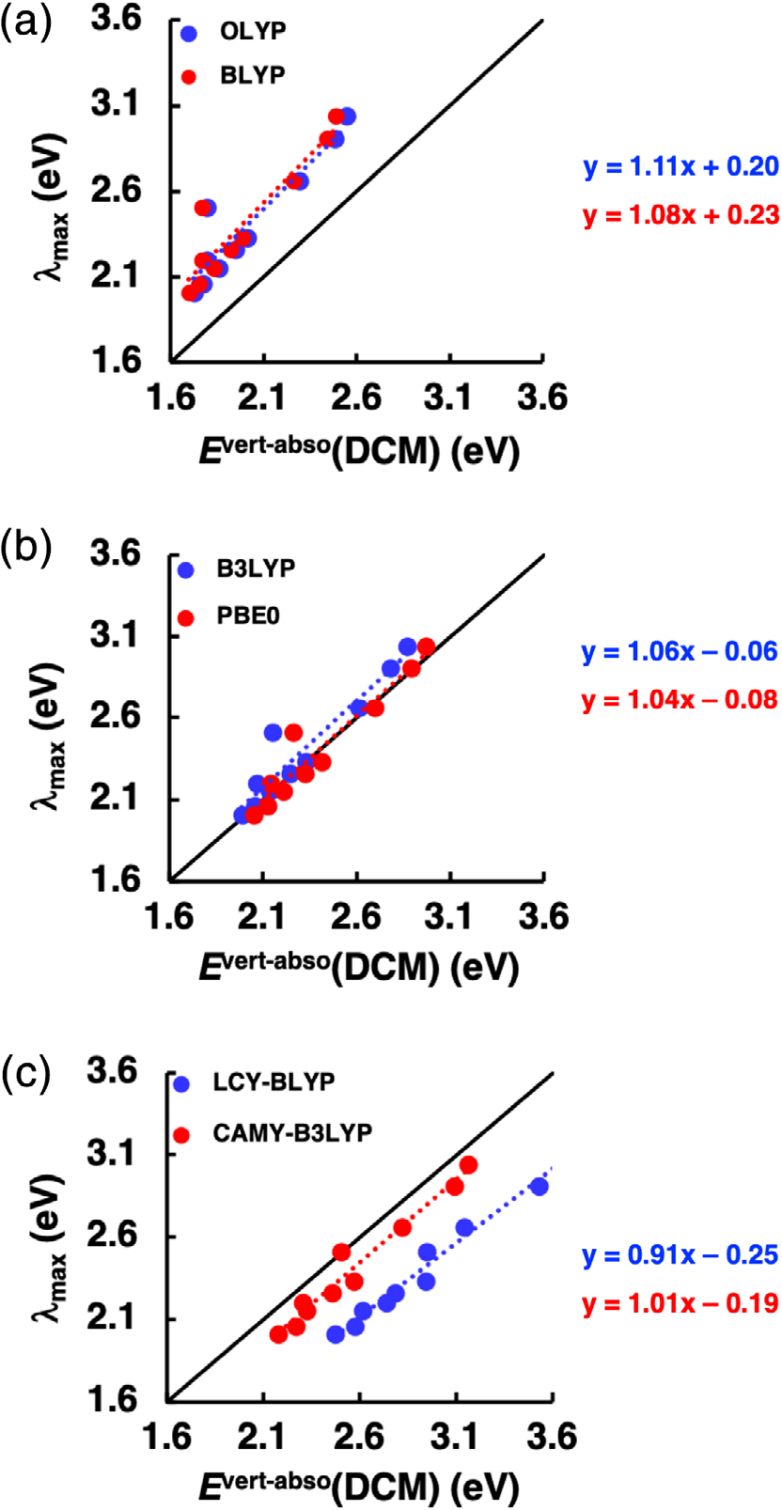
Accuracy plots of the lowest dipole‐allowed vertical excitation energies in DCM (*E* ^vert‐abso^(DCM)), computed at ZORA‐TDDFT/TZ2P//ZORA‐BP86/TZ2P using nonequilibrium COSMO, versus experimental λ_max_ values. The dotted lines denote a linear fit using simple linear regression [Color figure can be viewed at wileyonlinelibrary.com]

**Figure 3:**
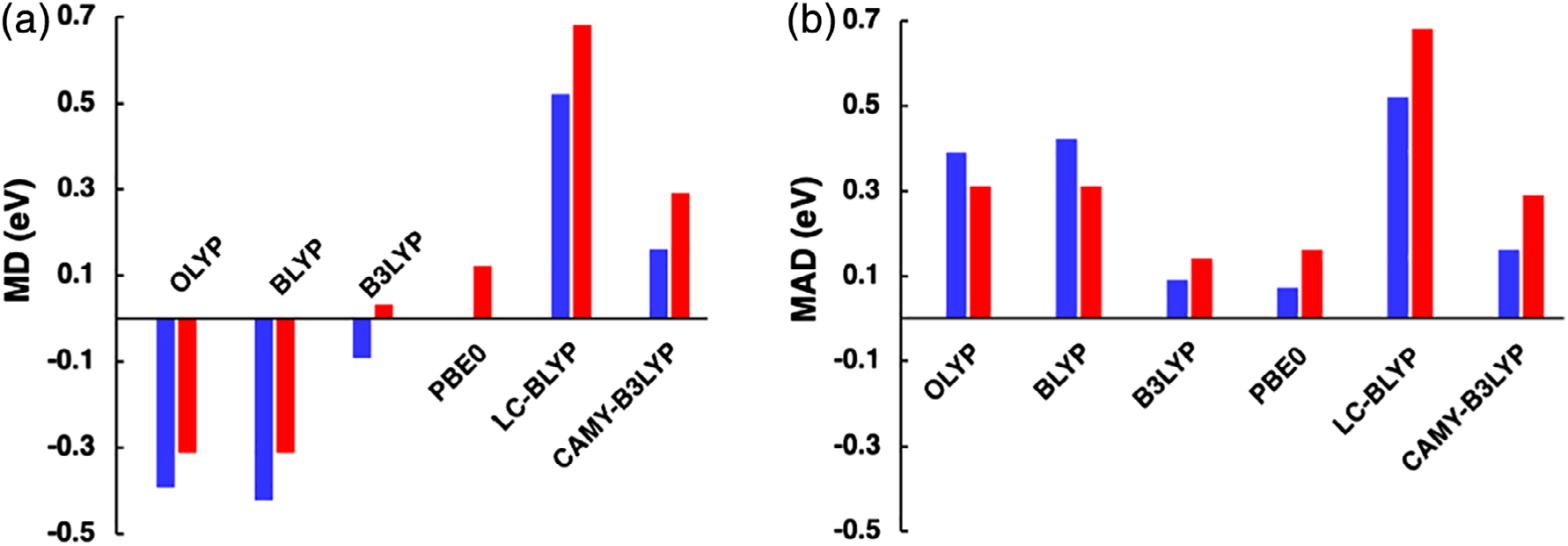
Mean absolute deviation (MAD) and mean deviation (MD) relative to experimental λ_max_ of the lowest dipole‐allowed vertical excitation energies of **1–10**, computed at ZORA‐TDDFT/TZ2P//ZORA‐BP86/TZ2P in gas‐phase (red) and in DCM solution (blue) using nonequilibrium COSMO [Color figure can be viewed at wileyonlinelibrary.com]

**Figure 4:**
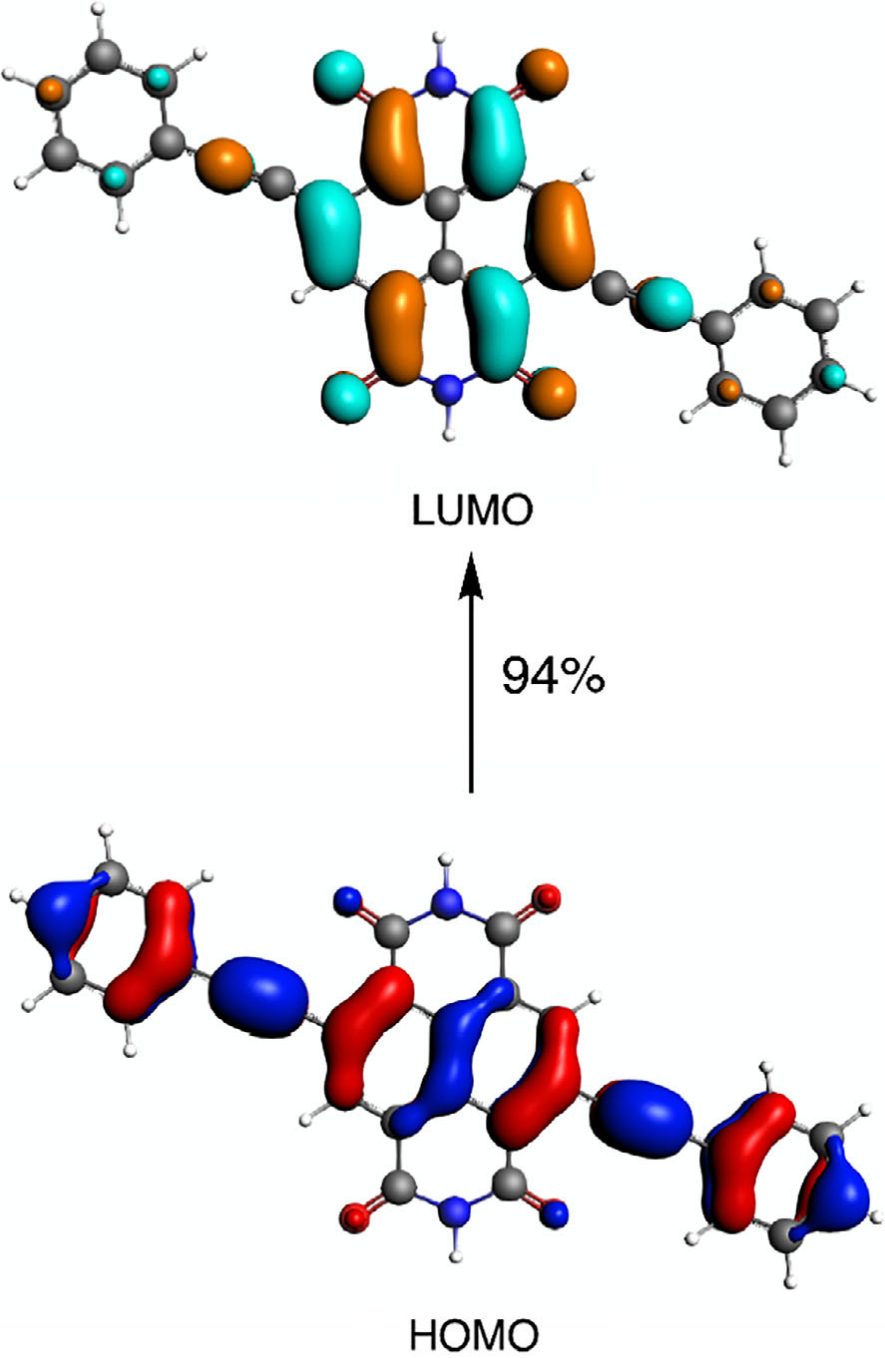
MOs involved in the lowest‐dipole allowed transition in **4**, computed at ZORA‐BLYP/TZ2P//ZORA‐BP86/TZ2P (plotted at isovalue ± 0.03 au) [Color figure can be viewed at wileyonlinelibrary.com]

The ability of various XC functionals to reproduce experimental λ_max_ was first assessed using statistical methods. The mean deviation (MD), mean absolute deviation (MAD) and maximum deviation (MAX) were calculated to discern the quantitative accuracy of the functionals. Additionally, correlation (R^2^) was also calculated to discern the systematic performance of the functional. These results are presented in Table 1 and Figures 2, 3, 4. Next, the performance of the OT‐RSH functional LC‐BLYP* was evaluated, results of which are presented in Table 2. Lastly, to obtain an optimal computational approach, the TDDFT data were linearly fitted and analyzed as summarized in Table 3.

**Table 1:** Statistics and error analysis of TDDFT functionals compared to experimental λ_max_ values for the lowest dipole‐allowed vertical excitation energy (*E* ^vert‐abso^(DCM), in eV) in dichloromethane calculated using the COSMO solvation model^a^

	GGA		GH		RSH	
Statistical parameters	OLYP	BLYP	B3LYP	PBE0	LCY‐BLYP	CAMY‐B3LYP
R^2^	0.86	0.86	0.90	0.92	0.98	0.96
MD	−0.39	−0.42	−0.09	0.00	0.52	0.16
MAD	0.39	0.42	0.09	0.07	0.52	0.16
MAX(+)^b^ (eV)	‐	‐	0.03	0.13	0.61	0.24
MAX(−)^b^ (eV)	−0.72	−0.74	−0.36	−0.25	‐	−0.01

a Computed at ZORA‐TDDFT/TZ2P//ZORA‐BP86/TZ2P using nonequilibrium COSMO for DCM solvation.

b Positive and negative MAX refer to the maximum overestimation and underestimation of λ_max_, respectively.

**Table 2:** Statistics and error analysis of the OT‐RSH LCY‐BLYP* compared to other XC functionals and experimental λ_max_ values for the lowest dipole‐allowed vertical excitation energy (*E* ^vert‐abso^(DCM), in eV) in dichloromethane calculated using the COSMO solvation model^a^

	GGA	GH	RSH		OT‐RSH
Statistical	BLYP	PBE0	CAMY‐B3LYP	LCY‐BLYP	LCY‐BLYP*
R^2^	0.86	0.92	0.96	0.98	0.96
MD (eV)	−0.42	0.00	0.16	0.52	0.11
MAD (eV)	0.42	0.07	0.16	0.52	0.12
MAX (+) (eV)^b^	‐	0.13	0.24	0.61	0.20
MAX (−) (eV)^b^	−0.74	−0.25	−0.01	‐	−0.05

a Computed at ZORA‐TDDFT/TZ2P//ZORA‐BP86/TZ2P using nonequilibrium COSMO for DCM solvation.

b Positive and negative MAX refers to the maximum overestimation and underestimation of λ_max_, respectively.

**Table 3:** Statistics and error analysis of *E* ^vert‐abso^(DCM) from linear fits of TDDFT data computed with various exchange‐correlation functionals to experimental λ_max_ values^a^

	GGA		GH		RSH		
Statistical parameters	OLYP	BLYP	B3LYP	PBE0	LCY‐BLYP	CAMY‐B3LYP	OT‐RSH LCY‐BLYP*
R^2^	0.86	0.86	0.90	0.92	0.98	0.96	0.96
MAD (eV)	0.09	0.08	0.07	0.07	0.04	0.04	0.05
MD (eV)	0.00	−0.01	−0.02	0.00	0.00	−0.02	−0.01
MAX (+) (eV)^b^	0.09	0.09	0.07	0.07	0.06	0.04	0.05
MAX (−) (eV)^b^	−0.35	−0.34	−0.29	−0.24	−0.07	−0.18	−0.16

a Computed using the SLR equations displayed in Figure 2 and section S1 of the Appendix S1.

b The positive and negative MAX refers to the maximum overestimation and underestimation of λ_max_, respectively.

Condensed‐phase TDDFT results are more accurate and systematic than those computed in the gas‐phase (except GGAs) (see Figure 3 and Tables S3‐S4, and Figure S2 in the Appendix S1) as experimental UV/Vis measurements were performed on samples dissolved in dichloromethane (DCM). All the condensed‐phase TDDFT excitation energies are red‐shifted compared to corresponding gas‐phase ones due to a larger stabilization of the excited state compared to the ground state. The electronic transition from the donor substituent (D) to the NDI acceptor (A) results in charge separation and a larger dipole moment in the excited state compared to the ground state, which is stabilized by the polar DCM solvent. Similar results were found in our previous study for D‐π‐A CT molecules.^72^ In light of these findings, we only discuss and compare the condensed‐phase excitation energies *E* ^vert‐abso^(DCM) with the measured λ_max_.

### 3.1 Condensed‐phase vertical excitation energies

The results of our statistical analyses of the excitation energies for the 10 systems calculated in the condensed‐phase are listed in Table 1 and visualized in Figures 2 and 3; the complete dataset is provided in Table S5 in the Appendix S1. Overall, PBE0 gives the best quantitative agreement with measured λ_max_ values, while LCY‐BLYP displays the most consistent systematic shift of λ_max_ values and thus has the highest predictive capability. The error in λ_max_ increases in the order GHs > RSHs > GGAs. We first discuss the quantitative agreement and then analyze the trends in the shift of the excitation energy of **1–10**.

The vertical excitations computed with the GHs are the most accurate ones of the three classes of functionals with MADs of 0.07 and 0.09 eV for PBE0 and B3LYP, respectively (see Figure 3). A moderate amount of exact exchange (20–25%) appears to be necessary to match the measured excitations of the cNDIs. PBE0 outperforms B3LYP in all statistical parameters (see Table 1) especially considering the fact that the errors are mostly negative for B3LYP than positive as in PBE0 (see Table S5 in the Appendix S1). A positive error sign reflects a better method than one giving a negative error if vibronic effects are to be accounted for, as pointed out by Ferrer et al.^43^ Namely, including vibronic effects would bring the overestimated PBE0 energies closer to the measured λ_max_ values, while the B3LYP energies would be further underestimated.

Among the RSHs, CAMY‐B3LYP shows a better quantitative agreement than LCY‐BLYP with MAD values of 0.16 and 0.52 eV, respectively (Figure 2c and Figure 3). The overestimation and the large disparity among the RSHs result primarily from their range separation parameter γ. By default, CAMY‐B3LYP and LCY‐BLYP are created with a γ of 0.34 and 0.75 *a*_0_^−1^, respectively. Following Equation (1), it would mean that a larger γ leads to a larger region in space where exact exchange is incorporated than DFT exchange. In addition, LCY‐BLYP contains the highest percentage of exact exchange (100% at long‐range) among the tested functionals. So, both, a larger γ and a larger fraction of exact exchange in LCY‐BLYP results in severely overestimated excitation energies.

In sharp contrast, the semi‐local GGAs severely underestimate the excitation energies (MAD ≈ 0.40 eV, see Figure 3), which concurs their well‐established findings for π‐conjugated systems.^73^ Such large underestimations stem primarily from an incorrect description of the delocalized or CT excitations, especially in the case of **4** (MAX = −0.72 eV). CT excitation energy in a D‐A system at asymptotically large distance *r* is given by

(3) $$E^{\mathrm{CT}}={\mathrm{IP}}^{D}–{\mathrm{EA}}^{A}–1/r$$

where 1/*r* term reflects the Coulomb attraction between the electron and the hole. Figure 4 depicts the spatial separation of the HOMO, which is delocalized over the extended π‐conjugated system, and the LUMO, which is localized on the NDI core, and is indicative of a CT excitation. The excitonic size parameter *d*_exc_ 74 derived from the interpretation of the one‐particle transition density matrix (1TDM) confirms the excitation of 4 over those of the other cNDIs to be CT dominated (see Table S6 in the Appendix S1 for details about *d*_exc_). The *d*_exc_ value of 6.47 Å for 4 is the longest of all the cNDIs, which concurs with the emergence of a CT error when GGAs are employed. This error in GGAs is primarily caused by two compounding factors, (a) the semi‐local nature of the exchange hole that leads to an unphysical stabilization of the nonlocal exchange hole for a CT excitation and (b) the underestimated orbital energy difference (which is the leading term in the TDDFT excitation energy) that should be close to IP – EA for a CT excitation (see Eq. (3)).^73^ As already discussed, ε(N) should be equal to –IP(N). However, the approximate GGA functional upshifts ε(N) due to the quadratic decay (1/*r* ^2^) of the exchange‐correlation potential instead of asymptotic behavior (1/*r*).^64^, ^73^, ^74^, ^75^ GHs partially mitigate this CT error by including a fraction of the exact exchange (–*a*_x_/*r*,) over the interelectronic distance resulting in a decrease of the error from –0.72 for OLYP to –0.25 eV for PBE0. That the CT error remains negative in sign indicates that a larger fraction of the exact exchange would be necessary to describe the CT excitation accurately. The smallest CT excitation error of 0.07 eV for 4 is obtained with the RSH functional CAMY‐B3LYP. This functional mitigates the underestimation of the CT energy by introducing a large fraction of exact exchange at long‐range together with a small fraction of exact exchange at short‐range to better describe the attractive electron‐hole interaction. This is further complemented by a balance between the DFT exchange and exact exchange at intermediate *r*. The result is in accord with past studies that highlighted the advantages of CAMY‐B3LYP to accurately reproduce electronic excitation spectra and response properties of strong CT D‐A systems.^76^, ^77^, ^78^, ^79^ So far, we have concentrated on accurately reproducing measured λ_max_ values, which led to a certain ranking of the tested functionals. However, such a ranking might obscure the relative performance of the functionals based on the systematic shift of λ_max_ for the cNDIs. For example, the MAD criteria indicates PBE0 to be the most accurate functional whereas the rather large variability of the computed excitation energies excitation energies might suggest otherwise. This system‐dependent behavior of PBE0 is reflected in the nonequivalent MD and MAD (see Table 1). In this respect, LCY‐BLYP shows, in fact, the best performance of all functionals (see Table 1). Its systematic and consistent overestimation of λ_max_ results in an excellent correlation of 0.98 with the experimental values. The primary reason for this behavior is that LCY‐BLYP treats valence and CT excitations on a similar footing. The excellent correlation found for LCY‐BLYP would mean that the computed excitation energy can be effectively calibrated to accurately predict λ_max_ of a new cNDI dye. The performance of CAMY‐B3LYP (R^2^ = 0.96) is nearly as good as LCY‐BLYP, but the GHs PBE0 (R^2^ = 0.92) and B3LYP (R^2^ = 0.90) are clearly not as good, with both GGAs being the worst (R^2^ = 0.86).

### 3.2 Effect of optimally‐tuned γ in LCY‐BLYP on vertical excitation energies

The statistical data for the performance of OT‐RSH LCY‐BLYP* are presented in Table 2 together with selected other functionals. The accuracy of the computed excitation energies improved with this optimized functional relative to GGAs and RSHs (PBE0 is still the most accurate) that also showed good predictability (the complete dataset is provided in Table S7 of the Appendix S1). The improvement in accuracy of LCY‐BLYP* over the standard LCY‐BLYP stems from a balanced DFT and exact exchange at short and long interelectronic distances. Optimal tuning of the γ parameter (see [Equations 1 and 2]) in LCY‐BLYP* reduces γ from its default value of 0.75 *a*_0_^−1^ to a range of 0.26–0.38 *a*_0_^−1^ depending on the particular cNDI (see Figure S3 in the SI). This large decrease in γ means that the change from DFT to exact exchange takes effect at longer inter‐electronic distances and implies more semi‐local DFT exchange than exact exchange (see Figure S4 in the SI). As a result, the excitation energies are lowered for the optimally tuned LCY‐BLYP*. Earlier studies reported that the inclusion of exact exchange at short‐range increased the accuracy of the computed excitation energies and polarizabilities.^64^, ^80^ To test this, we have performed additional calculations on compounds **1** and **2** with a modified LCY‐BLYP*, where the exact exchange was nonzero at short‐range, by setting *α* = 0.2 (*α* = 0.2, *β* = 0.8). At variance with the earlier studies, we found that the excitation energy actually increased for both **1** and **2** and led to a larger deviation from λ_max_ compared to the case where no exact exchange was used at short‐range (*α* = 0.0, *β* = 1.0, see Table S8 in the Appendix S1).

Although, the LCY‐BLYP* excitation energies are somewhat more accurate than those obtained with the standard RSHs and exhibit a better systematic shift than those obtained with the GHs, the relatively high computational price prohibits the use of this functional in a fast and predictive TDDFT protocol.

### 3.3 Calibration of condensed‐phase vertical excitation energy

Owing to the relatively good correlation exhibited by GHs and RSHs, the computed excitation energy can be calibrated to give a more accurate prediction of λ_max_ of a new cNDI molecule. In this regard, we have developed a tool based on fitting TDDFT computed excitation energies with experimentally determined λ_max_ values in DCM solution using simple linear regression (SLR) as shown in Figure 2. SLR entails calculation of a linear polynomial that determines the best fit between *E* ^vert‐abso^(DCM) and experimental λ_max_ value. The corresponding SLR polynomials are displayed in Figure 2 and section S1 in the Appendix S1. The statistical analysis of the calibrated data using the SLR equations for the various functionals is summarized in Table 3. The MDs and MADs improve substantially for the calibrated as compared to the original TDDFT values. This is so for all the functionals but the improvement is most pronounced in the case of RSHs. This is of course the direct consequence of the fact that the RSH functionals already showed the most superior correlation coefficient R^2^ between TDDFT and experimental data, and the fit basically eliminated the systematic error. The calibrated LCY‐BLYP method, represented by Equation (4), outperforms all other functionals regarding any statistical descriptor (see Table 3).

(4) $$\lambda_{\max}=-0.25+0.91E_{\mathrm{LCY}-\text{BLYP}}^{\text{vert}-\text{abso}}\;\left(\mathrm{DCM}\right)$$

This relationship constitutes a practical tool which, based on TDDFT computations at ZORA‐LCY‐BLYP/TZ2P in combination with the nonequilibrium COSMO bulk solvation model, predicts vertical excitation energies of an unknown cNDI with an accuracy of roughly ±0.04 eV, that is, within the “chemical accuracy” window of ±0.05 eV.^81^

## 4 CONCLUSION

We have explored and analyzed the performance of several exchange‐correlation functionals in gas‐ and condensed‐phase TDDFT computations to reproduce the experimental absorption energy maximum (λ_max_) of a set of 10 core‐substituted NDIs in DCM solution. The most accurate estimation of λ_max_ was obtained for the GH functional PBE0, whereas RSH LCY‐BLYP provides the most consistent systematic shift of excitation energy from λ_max_ upon introduction of various donor groups on the NDI core. Increasing the basis‐set size from TZ2P to QZ4P has only a negligible effect on the computed excitation energies. Inclusion of nonequilibrium COSMO bulk solvation is necessary to reproduce λ_max_ accurately.

The PBE0 functional shows the smallest mean absolute deviation of 0.09 eV and a considerable reduction of the charge‐transfer (CT) excitation error. However, the RSH functional CAMY‐B3LYP reproduces the CT excitation most accurately due to a balanced description of the short‐ and long‐range effects. The LCY‐BLYP functional, on the other hand, exhibits the most uniform error distribution leading to an excellent correlation of 0.98 between the computed excitation energies and observed λ_max_ values. The GGAs functionals significantly underestimate λ_max_ and are apparently unable to describe the CT excitations accurately.

The excellent correlation of LCY‐BLYP was leveraged to generate a calibrated variant based on a linear‐fit that gave the lowest mean absolute deviation of 0.04 eV. Self‐consistent tuning of the range‐separation parameter of the LCY‐BLYP functional gave only a minor improvement that does not merit the required significantly higher computational effort.

## Supporting information


**Appendix S1** Supporting Information.
