# Supplementary material for: Performance of TDDFT Vertical Excitation Energies of Core‐Substituted Naphthalene Diimides
Source: J Comput Chem. 2020 Mar 6;41(15):1448–55. doi: 10.1002/jcc.26188 (PMC7317478; doi:10.1002/jcc.26188)
Supplement: Supplementary file 1 — Appendix S1 Supporting Information. [file JCC-41-1448-s001.pdf]

Performance of TDDFT Vertical Excitation Energies of Core-Substituted Naphthalene Diimides

Ayush K. Narsaria,‡[a] Julian D. Ruijter,‡[a] Trevor A. Hamlin,[a] Andreas W. Ehlers,[b,c] Célia Fonseca Guerra,[a,d] Koop Lammertsma,*[a,c] and F. Matthias Bickelhaupt*[a,e]

[a] *Department of Theoretical Chemistry, Amsterdam Institute of Molecular and Life Sciences (AIMMS), and Amsterdam Center for Multiscale Modeling (ACMM), Vrije Universiteit Amsterdam, De Boelelaan 1083, 1081 HV Amsterdam, The Netherlands.*
E-mail: f.m.bickelhaupt@vu.nl, k.lammertsma@vu.nl

[b] *Van 't Hoff Institute for Molecular Sciences, University of Amsterdam, Science Park 904, 1098 XH Amsterdam, (The Netherlands).*

[c] *Department of Chemistry, University of Johannesburg, Auckland Park, Johannesburg, 2006 South Africa.*

[d] *Leiden Institute of Chemistry, Gorlaeus Laboratories, Leiden University, Leiden, The Netherlands.*

[e] *Institute of Molecules and Materials, Radboud University, Heyendaalseweg 135, 6525 AJ Nijmegen, The Netherlands.*

‡ Both authors contributed equally to this manuscript

Contents

Figure 1. Structure of molecule **1** as Full Molecule (left panel) as found in Ref. 1 and Simplified Molecule (right panel) where the phenyl rings at the diimide position has been replaced by hydrogen atoms.

Table S1. Comparison of the lowest dipole-allowed vertical excitation energy of compound **1** in the gas phase when phenyl rings at the diimide position are intact (full molecule) or replaced by –H (simplified molecule).

Table S2. Performance of basis set to reproduce experimental λ_{max} values for the lowest dipole-allowed vertical excitation energy, $E^{vert\text{-}abso}$ (in eV).

Table S3. Lowest dipole-allowed vertical excitation energies of compounds **1–10** in the gas-phase ($E^{vert\text{-}abso}$, in eV) calculated with various XC functionals. The experimental λ_{max} value is recorded in dichloromethane solvent.

Figure S2. Accuracy plots of the lowest dipole-allowed vertical excitation energies in gas-phase ($E^{\text{vert-abso}}$), computed at ZORA-TDDFT/TZ2P//ZORA-BP86/TZ2P, *versus* experimental λ_{max} values.

Table S4. Accuracy plots of the lowest dipole-allowed vertical excitation energies in gas-phase ($E^{\text{vert-abso}}$), computed at ZORA-TDDFT/TZ2P//ZORA-BP86/TZ2P, *versus* experimental λ_{max} values.

Table S5. Lowest dipole-allowed vertical excitation energies of compounds **1–10** in the condensed-phase ($E^{\text{vert-abso}}$(DCM), in eV) calculated with various XC functionals. The experimental λ_{max} value is recorded in dichloromethane solvent.

Table S6. Quantification of charge-transfer excitation using the excitonic distance parameter d_{exc} (in Å) and Tozer's index Λ.

Figure S3. Plot of the optimal tuning of the range-separation parameter γ in LCY-BLYP obtained by minimizing the *J* function for a representative set of cNDIs.

Figure S4. Partitioning of the Coulomb operator as a function of the range separation parameter γ at short-range (SR) and long-range (LR).

Table S7. Comparison of the performance of the lowest dipole-allowed vertical excitation energies ($E^{\text{vert-abso}}$(DCM), in eV) of compounds **1–10** obtained from OT-LCY-BLYP* with GGA-BLYP, RSH-CAMY-B3LYP and RSH-LCY-BLYP. The experimental λ_{max} value is recorded in dichloromethane solvent.

Table S8. Comparison of the performance of the lowest dipole-allowed vertical excitation energies ($E^{\text{vert-abso}}$(DCM), in eV) of compounds **1** and **2** obtained from OT-LCY-BLYP* when the fraction of exact exchange at short-range α is 0.0 or 0.2. The experimental λ_{max} value is recorded in dichloromethane solvent.

S1. Calibration Curves

Table S9. Cartesian coordinates (Å), energies, E_{Tot} (Hartree), and number of imaginary vibrational frequencies, N_{imag} of the optimized structures in the gas-phase obtained at ZORA-BP86/TZ2P level of theory.

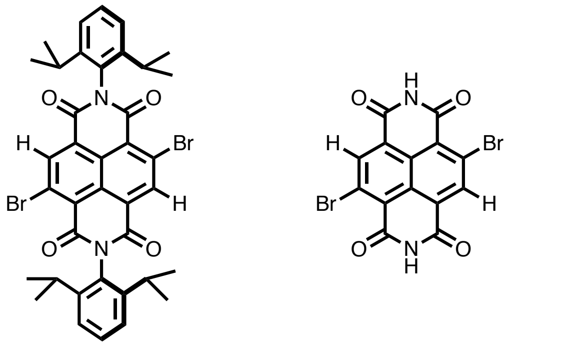

Figure S1. Structure of molecule **1** as Full Molecule (left panel) as found in Ref. 1 and Simplified Molecule (right panel) where the phenyl rings at the diimide position has been replaced by hydrogen atoms.

Table S1. Comparison of the lowest dipole-allowed vertical excitation energy of compound **1** in the gas phase ($E^{\text{vert-abso}}$, in eV) when phenyl rings at the diimide position are intact (full molecule) or replaced by –H (simplified molecule).[a]

Molecule	Full molecule			Simplified molecule		
	BLYP	PBE0	CAMY-B3LYP	BLYP	PBE0	CAMY-B3LYP
**1**	2.62	3.13	3.34	2.57	3.07	3.29

[a] Computed using ZORA-TDDFT/TZ2P//ZORA-BP86/TZ2P in gas-phase.

The lowest-dipole allowed excitation in gas phase ($E^{\text{vert-abso}}$) for **1** is almost the same for the full (with an alkyl or a phenyl chain) and the simplified molecule (the alkyl or the phenyl chain at the diimide position has been replaced by –H). $E^{\text{vert-abso}}$ changes by approximately 0.05 eV for the different classes of XC functional (Table S1). Therefore, the simplified model where the alkyl or the phenyl rings at the diimide positions have been replaced by –H can be used for further calculations.

**Table S2**. Performance of basis set to reproduce experimental λ_{max} values for the lowest dipole-allowed vertical excitation energy, $E^{vert-abso}$ (in eV).		
**Molecule**	**TZ2P**	**QZ4P**
**1**	2.57	2.58
**2**	2.48	2.50
**3**	2.39	2.39
**4**	1.84	1.88
**5**	2.14	2.14
**6**	2.07	2.07
**7**	1.84	1.84
**8**	1.96	1.94
**9**	1.91	1.91
**10**	1.85	1.84
R^2	0.77	0.82
MD (eV)	–0.30	–0.31
MAD (eV)	0.30	0.31
MAX (+) (eV)	-	-
MAX (–) (eV)	–0.67	–0.63

[a] Computed using ZORA-BLYP/TZ2P//ZORA-BP86/TZ2P in gas-phase
[b] The positive and negative MAX refers to the maximum overestimation and underestimation of λ_{max}, respectively.

**Table S3.** Lowest dipole-allowed vertical excitation energies of compounds **1–10** in the gas-phase ($E^{vert-abso}$, in eV) calculated with various XC functionals. The experimental λ_{max} value is recorded in dichloromethane solvent.[a]							
		**GGA**		**GH**		**RSH**	
**Molecule**	λ_{max}	**BLYP**	**OLYP**	**B3LYP**	**PBE0**	**LCY-BLYP**	**CAMY-B3LYP**
**1**	3.04	2.57	2.62	2.97	3.07	3.74	3.29
**2**	2.91	2.48	2.52	2.75	2.92	3.62	3.12
**3**	2.66	2.39	2.42	2.76	2.83	3.31	2.97
**4**	2.51	1.84	1.86	2.22	2.32	3.02	2.58
**5**	2.33	2.14	2.17	2.50	2.58	3.12	2.75
**6**	2.26	2.07	2.10	2.41	2.48	2.96	2.63
**7**	2.20	1.84	1.87	2.16	2.27	2.85	2.40
**8**	2.15	1.96	1.99	2.30	2.37	2.83	2.51
**9**	2.06	1.91	1.93	2.23	2.29	2.78	2.45
**10**	2.01	1.85	1.88	2.15	2.21	2.67	2.36

[a] Computed using ZORA-TDDFT/TZ2P//ZORA-BP86/TZ2P.

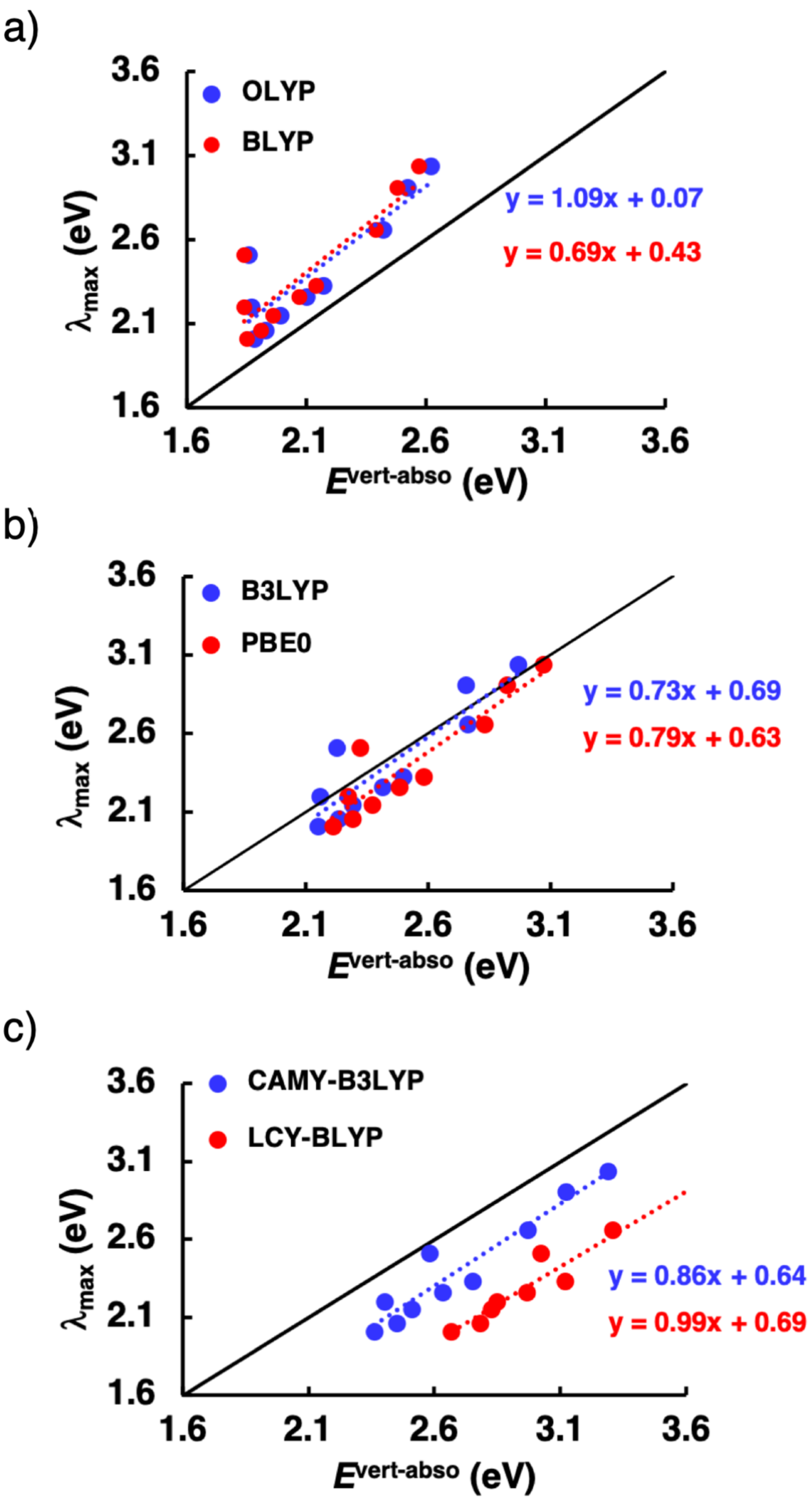

Figure S2. Accuracy plots of the lowest dipole-allowed vertical excitation energies in gas-phase ($E^{\text{vert-abso}}$), computed at ZORA-TDDFT/TZ2P//ZORA-BP86/TZ2P, *versus* experimental λ_{max} values. The dotted lines denote a linear fit using simple linear regression.

**Table S4.** Statistics and error analysis of various TDDFT functionals compared to experimental λ_{max} values for the lowest dipole-allowed vertical excitation energy ($E^{vert\text{-}abs}$, in eV) in gas-phase.[a]						
	**GGA**		**GH**		**RSH**	
**Statistical Parameters**	**OLYP**	**BLYP**	**B3LYP**	**PBE0**	**LCY-BLYP**	**CAMY-B3LYP**
R^2	0.78	0.78	0.80	0.85	0.96	0.91
MD (eV)	–0.28	–0.31	0.03	0.12	0.68	0.29
MAD (eV)	0.28	0.31	0.14	0.16	0.68	0.29
MAX (+) eV)[b]	-	-	0.17	0.25	0.79	0.42
MAX (–) eV)[b]	–0.65	–0.67	–0.29	–0.19	-	-

[a] Computed using ZORA-TDDFT/TZ2P//ZORA-BP86/TZ2P [b] The positive and negative MAX refers to the maximum overestimation and underestimation of λ_{max}, respectively.

**Table S5.** Lowest dipole-allowed vertical excitation energies of compounds **1–10** in the condensed-phase ($E^{vert\text{-}abso}$(DCM), in eV) calculated with various XC functionals. The experimental λ_{max} value is recorded in dichloromethane solvent.[a]							
		**GGA**		**GH**		**RSH**	
**Molecule**	λ_{max}	**OLYP**	**BLYP**	**B3LYP**	**PBE0**	**LCY-BLYP**	**CAMY-B3LYP**
**1**	3.04	2.55	2.49	2.87	2.97	3.61	3.16
**2**	2.91	2.48	2.44	2.78	2.89	3.53	3.09
**3**	2.66	2.29	2.26	2.61	2.70	3.14	2.82
**4**	2.51	1.79	1.77	2.15	2.26	2.95	2.50
**5**	2.33	2.01	1.98	2.33	2.41	2.94	2.57
**6**	2.26	1.95	1.92	2.25	2.32	2.78	2.46
**7**	2.20	1.80	1.77	2.07	2.14	2.74	2.31
**8**	2.15	1.86	1.83	2.13	2.21	2.62	2.33
**9**	2.06	1.77	1.75	2.06	2.12	2.58	2.27
**10**	2.01	1.72	1.70	1.99	2.05	2.47	2.18

[a] Computed using ZORA-TDDFT/TZ2P//ZORA-BP86/TZ2P.

To quantify the valence and charge-transfer nature of the excitations in **1–10** we evaluated the excitonic size parameter d_{exc} given by Plasser and coworkers[2] and also the Tozer's index Λ.[3] It can be clearly seen that Λ which is based on the spatial overlap of the canonical orbitals does not provide a clear picture of the different nature of excitations in **1–10**. This might be due to the extended π-framework of the systems studied, particularly in case of **4**.[4] However, d_{exc} which is derived from the interpretation of one particle transition density matrix (1TDM) as an excitonic wavefunction, delivers a better prediction of valence or charge-transfer excitations.

Table S6. Quantification of charge-transfer excitation using the excitonic distance parameter d_{exc} (in Å) and Tozer's index Λ.[a]

Molecule	d_{exc}	Λ
**1**	4.38	0.75
**2**	4.45	0.58
**3**	4.11	0.79
**4**	6.47	0.70
**5**	4.32	0.72
**6**	4.28	0.76
**7**	4.80	0.66
**8**	4.47	0.74
**9**	4.48	0.78
**10**	4.37	0.79

[a] The indexes are computed using ZORA-PBE0/TZ2P//ZORA-BP86/TZ2P.

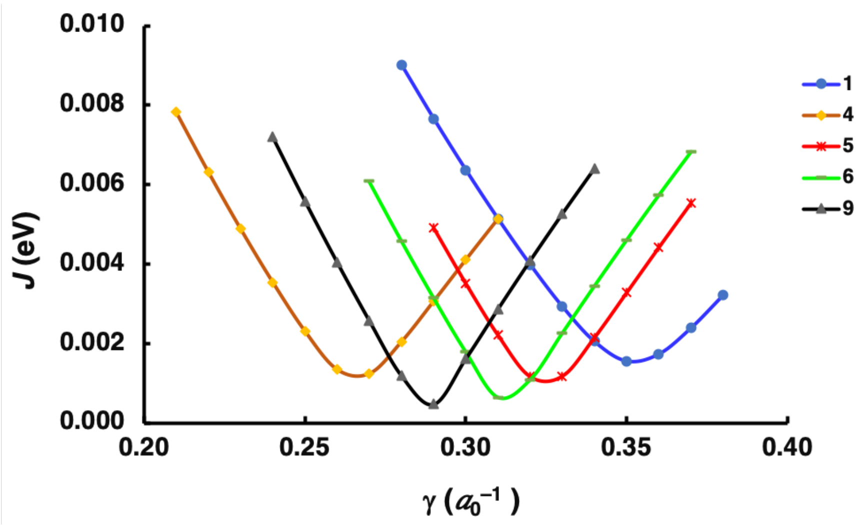

Figure S3. Plot of the optimal tuning of the range-separation parameter γ in LCY-BLYP obtained by minimizing the *J* function (see [Eqs. 1 and 2] in the main text) for a representative set of cNDIs.

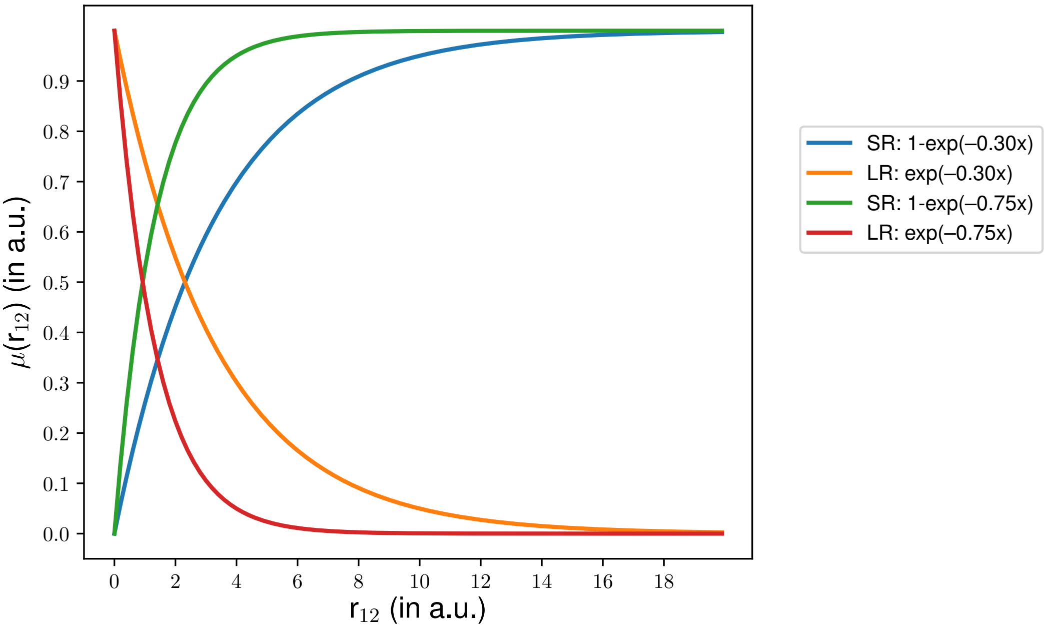

Figure S4. Partitioning of the Coulomb operator as a function of the range separation parameter γ at short-range (SR) and long-range (LR). See [Eq. (1)] in the article.

Table S7. Comparison of the performance of the lowest dipole-allowed vertical excitation energies ($E^{vert\text{-}abso}$(DCM), in eV) of compounds **1–10** obtained from OT-LCY-BLYP* with GGA-BLYP, RSH-CAMY-B3LYP and RSH-LCY-BLYP. The experimental λ_{max} value is recorded in dichloromethane solvent.[a]

			**GGA**	**GH**	**RSH**		**OT-RSH**
**Molecule**	λ_{max}	γ	**BLYP**	**PBE0**	**CAMY-B3LYP**	**LCY-BLYP**	**LCY-BLYP***
**1**	3.04	0.35	2.49	2.97	3.16	3.61	3.21
**2**	2.91	0.38	2.44	2.89	3.09	3.53	3.14
**3**	2.66	0.32	2.26	2.70	2.82	3.14	2.76
**4**	2.51	0.27	1.77	2.26	2.50	2.95	2.46
**5**	2.33	0.32	1.98	2.41	2.57	2.94	2.53
**6**	2.26	0.31	1.92	2.32	2.46	2.78	2.40
**7**	2.20	0.26	1.77	2.14	2.31	2.74	2.21
**8**	2.15	0.30	1.83	2.21	2.33	2.62	2.26
**9**	2.06	0.29	1.75	2.12	2.27	2.58	2.19
**10**	2.01	0.32	1.70	2.05	2.18	2.47	2.14

Table S8. Comparison of the performance of the lowest dipole-allowed vertical excitation energies ($E^{vert\text{-}abso}$(DCM), in eV) of compounds **1** and **2** obtained from OT-LCY-BLYP* when the fraction of exact exchange at short-range α is 0.0 or 0.2. The experimental λ_{max} value is recorded in dichloromethane solvent.[a]

Compound	λ_{max}	LCY-BLYP* (α = 0.0, β = 1.0)	LCY-BLYP* (α = 0.2, β = 0.8)
**1**	3.04	3.21	3.28
**2**	2.91	3.14	3.18

S1. Calibration Curves

$$\lambda_{max} = 0.23 + 1.08E^{\text{vert-abso}}_{\text{OLYP}}(\text{DCM}) \quad (3)$$

$$\lambda_{max} = 0.20 + 1.11E^{\text{vert-abso}}_{\text{BLYP}}(\text{DCM}) \quad (4)$$

$$\lambda_{max} = -0.06 + 1.06E^{\text{vert-abso}}_{\text{B3LYP}}(\text{DCM}) \quad (5)$$

$$\lambda_{max} = -0.08 + 1.04E^{\text{vert-abso}}_{\text{PBE0}}(\text{DCM}) \quad (6)$$

$$\lambda_{max} = -0.19 + 1.01E^{\text{vert-abso}}_{\text{CAMY-B3LYP}}(\text{DCM}) \quad (7)$$

$$\lambda_{max} = -0.25 + 0.91E^{\text{vert-abso}}_{\text{LCY-BLYP}}(\text{DCM}) \quad (8)$$

$$\lambda_{max} = 0.12 + 0.91E^{\text{vert-abso}}_{\text{LCY-BLYP*}}(\text{DCM}) \quad (9)$$

References

[1] S. Chopin, F. Chaignon, E. Blart, F. Odobel, *J. Mater. Chem.* **2007**, *17*, 4139.

[2] S. A. Mewes, J.-M. Mewes, A. Dreuw, F. Plasser, *Phys. Chem. Chem. Phys.* **2016**, *18*, 2548.

[3] M. J. G. Peach, P. Benfield, T. Helgaker, D. J. Tozer, *J. Chem. Phys.* **2008**, *128*, 044118.

[4] M. J. G. Peach, C. R. Le Sueur, K. Ruud, M. Guillaume, D. J. Tozer, *Phys. Chem. Chem. Phys.* **2009**, *11*, 4465.

Table S9. Cartesian coordinates (Å), energies, E_{Tot} (Hartree), and number of imaginary vibrational frequencies, N_{imag} of the optimized structures computed in the gas-phase at ZORA-BP86/TZ2P level of theory.

1
E_{Tot} = –6.84
N_{imag} = 0

C	-1.14261000	1.46706100	-0.06989600
C	-2.37483400	0.84718200	-0.04931300
C	-2.48369200	-0.55141100	0.01134400
C	-1.33687300	-1.35012400	0.05409900
C	2.37483400	-0.84718200	0.04931300
C	2.48369200	0.55141100	-0.01134400
C	1.33687300	1.35012400	-0.05409900
C	0.05763300	0.71010900	-0.03125700
C	-0.05763300	-0.71010900	0.03125700
C	1.14261000	-1.46706100	0.06989600
H	-3.27033800	1.46232700	-0.08050200
Br	-4.27451600	-1.20389800	0.02590200
C	-1.38750100	-2.83416500	0.12436900
H	3.27033800	-1.46232700	0.08050200
Br	4.27451600	1.20389800	-0.02590200
C	1.38750100	2.83416500	-0.12436900
C	1.10516500	-2.94464300	0.13268000
C	-1.10516500	2.94464300	-0.13268000
N	0.16071900	3.48878000	-0.15392100
N	-0.16071900	-3.48878000	0.15392100
O	-2.41538100	-3.50418800	0.15702600
O	2.10504000	-3.66040200	0.16532500
O	2.41538100	3.50418800	-0.15702600
O	-2.10504000	3.66040200	-0.16532500
H	0.21548500	4.50670500	-0.20022700
H	-0.21548500	-4.50670500	0.20022700

2
E = –6.63
N_{imag} = 0

C	1.249698	-1.416517	0.067739
C	2.455702	-0.712617	0.017115
C	2.455714	0.712599	0.017114
C	1.249714	1.416511	0.067743
C	-2.455700	0.712617	-0.017095
C	-2.455699	-0.712601	-0.017063
C	-1.249699	-1.416510	-0.067629
C	0.000004	-0.715011	0.000066
C	0.000014	0.715007	0.000066
C	-1.249688	1.416511	-0.067634
Br	4.129104	-1.605638	-0.080347
Br	4.129125	1.605596	-0.080377
C	1.233452	2.903235	0.224193
Br	-4.129126	1.605604	0.080222
Br	-4.129130	-1.605578	0.080309
C	-1.233437	-2.903239	-0.224050
C	-1.233409	2.903252	-0.223987
C	1.233422	-2.903250	0.224134
N	-0.000009	-3.491507	0.000035
N	0.000023	3.491504	0.000132
O	2.184163	3.609522	0.518710
O	-2.184110	3.609582	-0.518430
O	-2.184138	-3.609537	-0.518573
O	2.184124	-3.609560	0.518622
H	-0.000014	-4.511182	0.000016
H	0.000015	4.511180	0.000185000

3
E = –9.88
N_{imag} = 0

C	-1.67349300	0.89611200	-0.00012100
C	-2.55701300	-0.20596000	-0.00004300
C	-2.02152000	-1.51219500	-0.00010400
C	-0.66078100	-1.73647400	-0.00016600
C	2.55701400	0.20596100	-0.00003200
C	2.02152300	1.51219600	-0.00008700
C	0.66078300	1.73647600	-0.00015400
C	-0.26786600	0.66210400	-0.00015200
C	0.26786800	-0.66210300	-0.00015400
C	1.67349500	-0.89611100	-0.00012000
O	-3.88051400	0.02468300	0.00008400
H	-1.53692300	4.23953100	-0.00026000
C	-0.19445200	-3.14419600	-0.00021900
O	3.88051500	-0.02468000	0.00010100
H	1.53692000	-4.23953200	-0.00027400
C	0.19445200	3.14419800	-0.00020500
C	2.17669900	-2.29026000	-0.00017500
C	-2.17669900	2.29025900	-0.00016900
N	-1.18063700	3.28437500	-0.00021600
N	1.18063700	-3.28437500	-0.00022600
O	-0.94125600	-4.11754200	-0.00026600
O	3.34874900	-2.63868200	-0.00018400
O	0.94125300	4.11754600	-0.00024000
O	-3.34875000	2.63867900	-0.00018500
H	-2.66403000	-2.38756300	-0.00010000
H	2.66403400	2.38756300	-0.00007500
C	-4.79394400	-1.10011000	0.00028400
C	-6.19970700	-0.53502300	0.00065400
C	4.79394300	1.10011500	0.00030300
C	6.19970500	0.53502400	0.00064600
H	4.60895900	1.71531600	-0.89349200
H	4.60853700	1.71534000	0.89399700
H	-4.60897200	-1.71530100	-0.89352000
H	-4.60852800	-1.71534600	0.89396700
H	-6.37039300	0.08269100	0.89097800
H	-6.92541500	-1.35907400	0.00077500
H	-6.37081900	0.08280800	-0.88950600
H	6.37080700	-0.08277600	-0.88953700
H	6.37039400	-0.08272300	0.89094600
H	6.92541800	1.35907100	0.00079100

4
E = –13.17
N_{imag} = 0

C	-0.286968	0.780174	-1.435616

C	-0.520158	0.062665	-2.586590
C	-0.452871	-1.356066	-2.614924
C	-0.135848	-2.035395	-1.417747
C	0.660606	-1.243973	2.147788
C	0.592670	0.174733	2.176265
C	0.276251	0.854087	0.978938
C	0.034542	0.122660	-0.213832
C	0.106409	-1.303937	-0.225102
C	0.427921	-1.961447	0.996692
H	-0.762587	0.607220	-3.496553
C	-0.709488	-1.987811	-3.845076
N	-0.127891	2.898012	-0.288548
H	0.902832	-1.788547	3.057810
C	0.847447	0.806387	3.406850
C	0.192897	2.334979	0.953599
C	-0.378712	2.260688	-1.496606
C	-0.052660	-3.516291	-1.392381
N	0.268896	-4.079289	-0.150416
C	0.519715	-3.441985	1.057639
H	-0.183939	3.915896	-0.300278
H	0.324802	-5.097184	-0.138669
O	-0.649528	2.897773	-2.506430
O	0.380233	3.068096	1.915226
O	0.790527	-4.079082	2.067456
O	-0.240600	-4.249439	-2.353865
C	1.085673	1.234919	4.523900
C	1.343454	1.851302	5.769109
C	1.290828	3.260024	5.871548
C	1.542466	3.879061	7.092373
C	1.848398	3.111697	8.222807
C	1.902515	1.716229	8.129602
C	1.652689	1.085403	6.914017
H	1.051296	3.840044	4.981654
H	1.500441	4.965308	7.165524
H	2.044656	3.601231	9.176513
H	2.140533	1.119199	9.009230
H	1.692209	0.000394	6.830899
C	-0.950070	-2.416519	-4.961551
C	-1.211159	-3.033138	-6.205950
C	-1.519949	-2.267248	-7.350985
C	-1.773365	-2.898294	-8.565711
C	-1.723383	-4.293989	-8.657877
C	-1.417902	-5.061339	-7.527314
C	-1.162618	-4.442077	-6.307365
H	-1.556294	-1.182081	-7.268607
H	-2.011028	-2.301280	-9.445457
H	-1.922485	-4.783721	-9.610870
H	-1.379113	-6.147759	-7.599692
H	-0.923367	-5.022081	-5.417383

5

E = –8.60

N_{imag} = 0

C	2.082216	-0.558376	-0.000198
C	2.796758	0.651264	-0.002336
C	2.119249	1.874173	0.000127
C	0.735779	1.925407	0.003030
C	-2.214693	-0.424707	0.003370
C	-1.498711	-1.659605	0.007195
C	-0.127895	-1.700224	0.006547
C	0.660760	-0.509393	0.003241
C	-0.036180	0.734387	0.003756
C	-1.455852	0.782759	0.003968
Cl	4.533909	0.756263	-0.009431
H	2.380029	-3.896921	-0.001742
C	0.075803	3.244934	0.004304
H	-2.040296	-2.601283	0.009930
H	-1.789109	4.096989	0.004683
C	0.527177	-3.028545	0.007579
C	-2.121127	2.073987	0.003164
C	2.748604	-1.886096	-0.003004
N	1.904577	-2.994081	-0.000965
N	-1.304608	3.199172	0.004107
O	0.674025	4.324221	0.005026
O	-3.358565	2.238242	0.001766
O	-0.078760	-4.100113	0.013949
O	3.962114	-2.073260	-0.006779
H	2.681345	2.804595	-0.000984
N	-3.558040	-0.418972	-0.001307
C	-4.414412	-1.604944	-0.002514
C	-5.881676	-1.195362	-0.025571
H	-3.974930	0.519029	-0.001409
H	-4.201545	-2.209802	0.891399
H	-4.178330	-2.224719	-0.880209
H	-6.515358	-2.089951	-0.025170
H	-6.114788	-0.611616	-0.926415
H	-6.138276	-0.594522	0.857486

6

E = –11.27

N_{imag} = 0

C	0.092221	1.543909	-1.014269
C	0.174108	0.966225	-2.312432
C	0.135821	-0.454280	-2.408655
C	0.024608	-1.253529	-1.297277
C	-0.253056	-0.941782	2.448959
C	-0.216963	0.461176	2.555090
C	-0.105416	1.267895	1.436494
C	-0.022745	0.713534	0.134175
C	-0.059013	-0.707073	0.019212
C	-0.173351	-1.537217	1.166411
N	0.284267	1.732728	-3.417150
H	0.194275	-0.947537	-3.375536
N	0.039804	3.465161	0.457636
O	-0.361034	-1.743590	3.525491
H	-0.275356	0.958307	3.518399
C	-0.074193	2.733182	1.633877
C	0.125650	2.989598	-0.849706
C	-0.006822	-2.723412	-1.504768
N	-0.118835	-3.456482	-0.338340
C	-0.206371	-3.009032	0.993564
H	0.064257	4.479030	0.558745
H	-0.142749	-4.470030	-0.444401
O	0.223593	3.813045	-1.777181
O	-0.140565	3.292398	2.724075
O	-0.300356	-3.843573	1.882003
O	0.058936	-3.268326	-2.601572
C	0.590007	1.929346	-7.222595

H	-0.384869	3.057064	-5.648927
H	-0.514996	0.630449	-5.022314
H	1.250901	0.572948	-4.883642
H	1.376863	3.000084	-5.510036
C	0.375209	1.235533	-4.782563
C	0.490386	2.397634	-5.767155
H	0.301698	2.742381	-3.223864
C	0.705506	3.091856	-8.212824
H	-0.294442	1.320252	-7.469571
H	1.461416	1.263680	-7.331156
C	-0.442693	-1.142337	4.839173
C	-0.555322	-2.272176	5.842431
H	-1.321447	-0.480372	4.880519
H	0.459371	-0.536708	5.016969
H	0.321173	-2.929441	5.787558
H	-1.452922	-2.873184	5.651757
H	-0.619805	-1.856911	6.856816
H	1.599357	3.698936	-8.009300
H	-0.168505	3.755858	-8.148641
H	0.775367	2.729913	-9.247020

7
E = –12.28
N_{imag} = 0

C	1.122201	1.566924	-0.243145
C	2.386850	0.952316	-0.282439
C	2.469928	-0.399879	0.189127
C	1.314218	-1.209039	0.126527
C	-2.428397	-0.760040	-0.176710
C	-2.516379	0.639974	0.115674
C	-1.355938	1.430653	0.046717
C	-0.069745	0.806830	-0.056979
C	0.024391	-0.614753	-0.031356
C	-1.174064	-1.393620	-0.149630
S	3.806271	1.843168	-0.827532
H	-0.317320	4.558055	-0.473940
C	1.433474	-2.678184	0.101856
S	-3.982648	-1.528571	-0.528356
H	0.298792	-4.373900	0.043532
C	-1.456994	2.896257	-0.135121
C	-1.070976	-2.865636	-0.063783
C	1.036084	3.028025	-0.371224
N	-0.248691	3.547442	-0.362035
N	0.225249	-3.357785	0.030748
O	2.478867	-3.326353	0.097250
O	-2.002851	-3.666480	-0.036881
O	-2.491004	3.559939	-0.151285
O	2.002527	3.782658	-0.465257
S	3.988231	-0.860697	0.945840
S	-4.141422	1.211315	0.502685
C	4.861321	0.542157	-1.625257
C	5.569018	1.198262	-2.811271
C	-3.668908	-2.544075	-2.035628
C	-4.971158	-2.645544	-2.827613
H	-2.880844	-2.043379	-2.612208
H	-3.305173	-3.523471	-1.709839
H	5.585989	0.152155	-0.902025
H	4.214450	-0.281602	-1.949377
H	4.851551	1.538122	-3.568744
H	6.252098	0.474797	-3.277810
H	6.166221	2.064241	-2.494578
H	-5.314327	-1.664796	-3.179711
H	-5.772106	-3.095974	-2.225677
H	-4.811772	-3.291274	-3.702353
C	3.534662	-1.965055	2.339831
C	4.522668	-1.728873	3.481316
C	-3.906715	2.324683	1.954761
C	-5.124543	2.197104	2.867856
H	2.505944	-1.716208	2.633232
H	3.553603	-2.997904	1.977080
H	4.460745	-0.704224	3.868064
H	5.557261	-1.914555	3.162355
H	4.300017	-2.424512	4.301994
H	-2.984027	2.015571	2.461862
H	-3.774275	3.342283	1.574829
H	-5.223572	1.184443	3.278201
H	-6.053073	2.444988	2.335699
H	-5.024700	2.902372	3.704777

8
E = –11.68
N_{imag} = 0

C	-0.293477	1.774637	-0.032538
C	-1.613796	1.359349	-0.023973
C	-1.944574	-0.014652	-0.003460
C	-0.873821	-0.967192	0.007774
C	2.903339	-1.037715	-0.007073
C	3.200876	0.377508	-0.000701
C	2.139909	1.298314	-0.016229
C	0.782736	0.854196	-0.021444
C	0.480554	-0.534908	0.001378
C	1.561108	-1.473587	0.006213
C	-6.931559	0.545549	0.001629
H	1.533354	4.570413	-0.012552
C	-1.168682	-2.389839	0.018937
N	3.935819	-1.915481	-0.020852
H	-0.273984	-4.236222	0.007618
C	2.428081	2.727362	0.000946
C	1.277251	-2.899641	-0.003063
C	-0.025707	3.230257	-0.042045
N	1.325653	3.572712	-0.016624
N	-0.063953	-3.239250	0.008273
O	-2.303790	-2.900983	0.032336
O	2.131596	-3.809845	-0.020733
O	3.567733	3.228846	0.030811
O	-0.886439	4.103480	-0.063872
N	-3.234782	-0.424321	0.005759
N	4.505228	0.765523	0.008504
H	-5.702968	-1.027500	-0.843177
C	5.568201	-0.159539	0.362665
H	-4.349424	1.143209	0.857237
H	-4.361765	1.096763	-0.913202
H	-5.692410	-0.975117	0.923819
C	-5.683483	-0.342946	0.020738
C	-4.385508	0.462086	-0.010406
C	-8.234320	-0.259107	0.038141
H	-8.307367	-0.934934	-0.826305
H	-8.294140	-0.875606	0.946818
H	-9.112606	0.399982	0.022656

H	-2.386110	2.122911	-0.030883
H	-6.913282	1.178103	-0.900652
H	-6.899556	1.235398	0.860522
C	5.300537	-1.503742	-0.302284
H	-3.355114	-1.443902	0.020110
H	5.631163	-0.293408	1.456442
H	6.523041	0.252206	0.015114
H	5.984737	-2.266019	0.089356
H	5.468249	-1.420187	-1.389434
H	3.631890	-2.901028	-0.071920
H	4.624892	1.784999	0.082203

9
E = –12.09
N_{imag} = 0

C	0.578508	-1.741149	-0.220203
C	1.936018	-1.569030	-0.351400
C	2.580202	-0.305048	-0.216744
C	1.710463	0.835938	-0.181798
C	-1.936003	1.569052	0.351339
C	-2.580184	0.305066	0.216740
C	-1.710445	-0.835918	0.181760
C	-0.301150	-0.644232	0.000386
C	0.301168	0.644250	-0.000405
C	-0.578491	1.741175	0.220129
H	2.509316	-2.474037	-0.520783
N	3.942391	-0.296799	-0.086659
N	-1.259707	-3.232130	0.125835
H	-2.509307	2.474067	0.520660
N	-3.942387	0.296811	0.086725
C	-2.150901	-2.205705	0.474457
C	0.068898	-3.130254	-0.244633
C	2.150917	2.205703	-0.474577
N	1.259729	3.232145	-0.125992
C	-0.068882	3.130285	0.244451
H	-1.608986	-4.173395	0.300437
H	1.609000	4.173401	-0.300653
O	0.741774	-4.124636	-0.502483
O	-3.204464	-2.532728	1.028466
O	-0.741755	4.124680	0.502253
O	3.204462	2.532695	-1.028643
C	6.098853	-1.211721	0.424387
C	4.731054	-1.550234	-0.184755
C	6.193504	0.312097	0.290960
C	4.756019	0.772607	0.519505
C	-4.731065	1.550220	0.185004
C	-6.099013	1.211665	-0.423801
C	-6.193504	-0.312182	-0.290553
C	-4.756038	-0.772516	-0.519545
H	6.913192	-1.748358	-0.076707
H	6.112802	-1.497517	1.485664
H	4.807715	-1.857413	-1.239322
H	4.248380	-2.365523	0.369538
H	6.510719	0.593310	-0.723597
H	6.891429	0.767792	1.003436
H	4.543222	1.732806	0.043063
H	4.516459	0.846074	1.596134
H	-4.807457	1.857407	1.239590
H	-4.248572	2.365530	-0.369424
H	-6.113284	1.497590	-1.485043
H	-6.913258	1.748172	0.077586
H	-6.891576	-0.767842	-1.002909
H	-6.510412	-0.593570	0.724051
H	-4.516731	-0.845661	-1.596253
H	-4.543078	-1.732818	-0.043394

10
E = –10.31
N_{imag} = 0

C	-1.673504	0.873761	0.000103
C	-2.572701	-0.233373	0.000106
C	-2.013332	-1.540033	0.000163
C	-0.650624	-1.745477	0.000135
C	2.572701	0.233334	-0.000031
C	2.013330	1.539989	0.000025
C	0.650628	1.745430	0.000071
C	-0.270396	0.657732	0.000086
C	0.270403	-0.657780	0.000079
C	1.673513	-0.873804	0.000020
N	-3.912738	-0.050572	0.000034
H	-1.602517	4.204797	0.000127
C	-0.154965	-3.141840	0.000167
H	2.652782	2.418952	0.000022
H	1.602543	-4.204838	0.000119
C	0.154984	3.141796	0.000095
C	2.184007	-2.238194	0.000022
C	-2.183985	2.238158	0.000113
N	-1.230159	3.256021	0.000115
N	1.230183	-3.256061	0.000113
O	-0.871081	-4.137807	0.000231
O	3.386839	-2.553215	-0.000050
O	0.871107	4.137757	0.000105
O	-3.386813	2.553200	0.000108
H	-2.652786	-2.418995	0.000230
N	3.912744	0.050588	-0.000137
C	4.900441	1.119739	-0.000128
C	6.310750	0.539640	-0.000546
H	4.203079	-0.934502	-0.000139
H	4.757351	1.760539	0.885470
H	4.756987	1.760879	-0.885418
H	7.051517	1.348360	-0.000396
H	6.481837	-0.079726	-0.891484
H	6.482131	-0.080315	0.889928
C	-4.900531	-1.119629	0.000034
C	-6.310795	-0.539387	-0.000915
H	-4.203009	0.934539	0.000073
H	-4.756910	-1.760972	-0.885079
H	-4.757747	-1.760260	0.885807
H	-6.482391	0.080816	0.889357
H	-7.051637	-1.348039	-0.000778
H	-6.481565	0.079778	-0.892056
